# The Feasibility of an Educational Course for General Practitioners on Otolaryngologic Morbidity

**DOI:** 10.7759/cureus.65171

**Published:** 2024-07-23

**Authors:** Nikolaos Papadopoulos, Alexander D Karatzanis, Emmanuel P Prokopakis, Manolis Linardakis, Ioannis Galanos, Georgia Liva, Ioannis Tsamandouras, Evangelia Mourellou, Emmanouil K Symvoulakis

**Affiliations:** 1 Social and Family Medicine, University of Crete, Heraklion, GRC; 2 Otorhinolaryngology - Head and Neck Surgery, University of Crete, Heraklion, GRC; 3 Social Medicine, University of Crete, Heraklion, GRC

**Keywords:** otolaryngology, questionnaire, seminar, primary care, general practitioner, ent

## Abstract

Objective

To test feasibility by enhancing the knowledge and skills of general practitioners (GPs) in managing ear, nose, and throat (ENT) morbidity within primary care settings through a three-hour educational course.

Methods

A structured course focused on common ENT disorders was conducted. Case morbidity was selected based on appropriate criteria. The participants (n=34) were GPs randomly assigned to control and intervention groups. A questionnaire assessed knowledge, attitudes, and practices (KAP) before and after the course using proper analysis.

Results

The intervention group showed significant improvement in responses within five of sixteen questions (p<0.05). Participants demonstrated greater knowledge responsiveness in relation to epistaxis and CENTOR criteria, while knowledge response improvement was poor in regard to vestibular morbidity. Overall improvement in KAP scores (p<0.05), with high effect sizes, was achieved before and after the seminar.

Conclusions

The three-hour ENT course for GPs was found to be feasible, emphasizing the need for targeted short-duration courses within GP education supported by locally relevant information on common ENT conditions. Future research should explore the long-term impact of similar collaborative interventions in primary care.

## Introduction

Primary healthcare offers promising potential to support healthcare systems in an effort to alleviate hospital burden. Existing literature already suggests that major socioeconomic benefits may arise by shifting from a hospital-based setting to primary care facility services [1-2]. Consequently, many countries have substantially directed resource investment and introduced favorable policies for strengthening their core [3]. In every successful primary healthcare system, the role of primary care physicians (PCPs) as the first point of contact for patients is well acknowledged. They serve as contact facilitators and decision-makers, handling a miscellaneous array of medical scenarios, from managing chronic conditions to treating acute care events. While managing chronic conditions should be unarguably their main practice competency, efficient management of many acute conditions can further lead to workload reduction, as well as resource spending decrease within hospital emergency departments [1,2].

Ear, nose, and throat (ENT) symptoms and problems are quite common in all communities and demand both a precise diagnosis and a rapid intervention to prevent serious complications [4]. These clinical entities encompass a broad spectrum of conditions, ranging from life-threatening airway obstruction to epistaxis and acute tonsilitis, each necessitating distinct treatment and prompt responses. The management of ENT emergencies presents a unique challenge for PCPs, as these situations often require specialized skills and knowledge that may extend, to some degree, beyond their usual scope of practice. While immediate referral to specialists is an option, it may not always be feasible due to various factors such as geographic limitations, patient preferences, and resource availability [5]. Therefore, focused training of PCPs with competence and confidence might enable them to identify, assure, and initiate appropriate interventions for common ENT morbidity, thus ensuring timely and effective patient care.

Traditionally, general practitioner (GP) education should be composed of adequate amounts of empirical skills mixed with evidence-based scientific knowledge during undergraduate and postgraduate training. This becomes more evident in clinical fields where outcomes equally depend on both components. As ENT morbidity highly requires both clinical and practical skills, it is demanding, as well. A study in the UK reported low confidence rates among GPs when called to treat ENT cases [6]. In Greece, the curriculum of General and Family Medicine residency, as of 2019, includes just a few months of training in otorhinolaryngology, ophthalmology, urology, and orthopedic outpatient clinics, while PCPs back in the 1990s were mainly trained in internal medicine and surgery. Consequently, many GPs may have different but sound reasons to pursue continuous education, as a need and not a luxury, to confidently deal with ENT acute morbidity.

Training and guidance from hospital specialists have been shown to have a positive impact on making GP trainees better skilled to provide medical treatment through increased confidence in their own knowledge [6]. The purpose of this study is to examine the feasibility of organizing an educational seminar, aiming to show whether it is needed and how to improve the knowledge and skill set of qualified GPs in treating ENT morbidity in primary care settings. Testing a three-hour course on the most common ENT problems, as emerged recently from large-scale local data, will assist in answering the above-mentioned research questions.

## Materials and methods

In the context of this investigation, a carefully structured three-hour intensive educational course on ENT morbidity, commonly encountered in the local community, was organized. This educational initiative was designed to address the specific challenges and dilemmas posed by ENT case management, aiming to enhance the knowledge and skills of GPs working in primary care settings in the county of Heraklion, Crete, Greece.

Selection criteria of the ENT cases

The inclusion criteria for the clinical themes applied in the course were thoroughly defined, considering factors such as increased prevalence, referral rate to hospitals, low admission frequency, and the feasibility of treating these conditions with non-specialized equipment available in primary care settings. The selection process was informed by a study conducted at the University General Hospital of Heraklion and published in 2023, identifying the most common ENT problems encountered in the emergency department (ED) [7].

The prevalent ENT problems observed in the above-mentioned study included otitis externa, epistaxis, impacted cerumen, otitis media, benign paroxysmal positional vertigo, vestibular neuritis, acute pharyngitis, and acute tonsillitis. These clinical conditions, as expected and also as shown by the study, led mostly to low hospitalization decisions and management within outpatient services without any need for admission [7]. Impacted cerumen was not included, as it did not endorse an urgent clinical priority for referral decisions. Severe complications, such as mastoiditis (linked to acute otitis media) and peritonsillar abscess (associated with acute tonsillitis), showed high admission rates and were intentionally selected as topics. Additionally, acute airway obstruction was included due to the urgency of the condition. To enhance the educational completeness and usefulness of the training material, the eligible themes, based on the described theoretical framework, were systematically grouped into five sections as follows: 1) otitis externa and otitis media with a detailed reference to mastoiditis as a complication; 2) acute tonsillitis with a detailed reference to peritonsillar abscess as a complication; 3) benign paroxysmal positional vertigo and vestibular neuritis; 4) epistaxis; 5) acute airway obstruction.

Participant recruitment and course delivery

The training course was materialized through a lecture format with useful additional audiovisual aids, as their utilization has been proven to assist in the assimilation of knowledge [8]. The lectures were facilitated by two experienced ENT academics from the University of Crete with the aid of ENT residents of the University General Hospital of Heraklion. The collaboration between the Clinic of Social and Family Medicine and the Department of Otorhinolaryngology, spanning decades, underscores a history of successful interdepartmental initiatives and research [7,9,10].

GPs from the county of Heraklion in Crete, working at public primary care units, were invited to attend the educational course. Of a total of 103 GPs active within public primary care settings in the county, 51 agreed to participate after written informed consent. To ensure an unbiased assessment, initial randomization targeted 21 practitioners for each of the two groups. Due to last-minute denials, 17 participants with physical attendance at a welcome lunch lecture hall were included in the intervention group, being matched with the 17 colleagues forming the control group. Finally, the lectures with the supplementary audiovisual material used were later sent to all 51 GPs who agreed to participate in the study.

Questionnaires

To assess the effectiveness of the course, comprehensive questionnaires were developed, encompassing three key domains: knowledge, attitudes, and practices (KAP). The questionnaire structure involved nine questions pertaining to knowledge, four questions addressing attitudes, and three questions focusing on practices.

Within the knowledge section, participants were additionally prompted with the query: "How certain are you of your answer?" for each of the nine questions. The response options ranged from 1 indicating "not at all" to 5 indicating "absolutely."

The questionnaires were administered at two distinct time points: prior to the beginning of the course and immediately after its completion. All questions adhered to a multiple-choice question (MCQ) format, aligning with disease-oriented guidelines whenever applicable, and in instances where specific guidelines were unavailable, responses were derived from existing literature [11-25].

Across all three sections, questions covered various aspects, including four related to epistaxis, three addressing vertigo-related ENT problems, four concerning otitis externa and acute otitis media, four focusing on upper respiratory infections (URIs), such as acute tonsillitis, and one delving into anaphylaxis.

Statistical analysis

Data were analyzed using the SPSS software version 25.0 (IBM Inc., Armonk, New York). Frequencies and mean levels of descriptive characteristics of the 34 physicians were estimated according to the two groups of intervention and control and using binomial, χ^2^ (Chi-square), and Student t-tests to compare any difference. Differences in correct responses in the intervention group between pre-post intervention were assessed with the McNemar test while in certainty levels and in correct responses (scores 0-100), Mann-Whitney and Wilcoxon sign rank tests were used. The effect size (η^2^ or |r|) was also estimated according to the formula of r=Z/√n to assess the degree of score changes in pre-post levels. A critical value of 5% was selected as the level of significance.

Ethical approval

This study received ethical approval from the Seventh Health Region of Greece (approval number 26459/19-06-23) and the Ethics Committee of the University of Crete (approval number 59/05-05-2021). The research was conducted ethically, with all study procedures being performed in accordance with the requirements of the Declaration of Helsinki. Written informed consent was obtained from each participant in the study.

## Results

The descriptive characteristics of both groups are presented in Table 1. Among the 34 participants in both groups, 18 were men and 16 were women, while the mean age of the two groups was 48.6 years for the intervention group (IG) and 49.1 years for the control group (CG). In the IG, participants were working as specialists for a mean period of 14.2 years, while in the CG, the mean period was 17.0 years.

**Table 1:** Descriptive characteristics of 34 physicians in two groups: intervention and control χ^2^ and Student t-tests - there were no significant differences observed at a p-value <0.05

Particulars		Groups	
		Intervention (n=17)	Control (n=17)
		n (%)	n (%)
Sex	Male	10 (58.8)	8 (47.1)
	Female	7 (41.2)	9 (52.9)
Age, years	mean±SD	48.6±8.4	49.1±8.7
Time working as a specialist, years	mean±SD	14.2±7.6	17.0±7.5
Postgraduate education	MSc	8 (47.1)	8 (47.1)
	PhD	1 (5.9)	3 (17.6)
Certification of English language	Yes	16 (94.1)	12 (70.6)
Use of internet for searching medical information	Never	-	1 (5.9)
	Sometimes	-	1 (5.9)
	Often	9 (52.9)	7 (41.2)
	Everyday	8 (47.1)	8 (47.1)
Workplace	Health Center outside the city of Heraklion	13 (76.5)	11 (64.7)
	Health Center inside the city of Heraklion	2 (11.8)	2 (11.8)
	Satellite practice	2 (11.8)	4 (23.5)
International work experience	Yes	4 (23.5)	1 (5.9)

In both groups, half reported using the internet on a daily basis in research of medical knowledge, and most participants were employed in rural primary health centers, accounting for 76.5% in the IG and 74.7% in the CG. Moreover, 17.6 % of the participants of the CG had a PhD, while for the IG only 5.9% had such a qualification. However, 94.1% of the IG had a certification in English language compared to 70.6 for the CG, and 23.5% of the IG had international work experience in comparison to 5.9% of the CG. 

**Figure 1:**
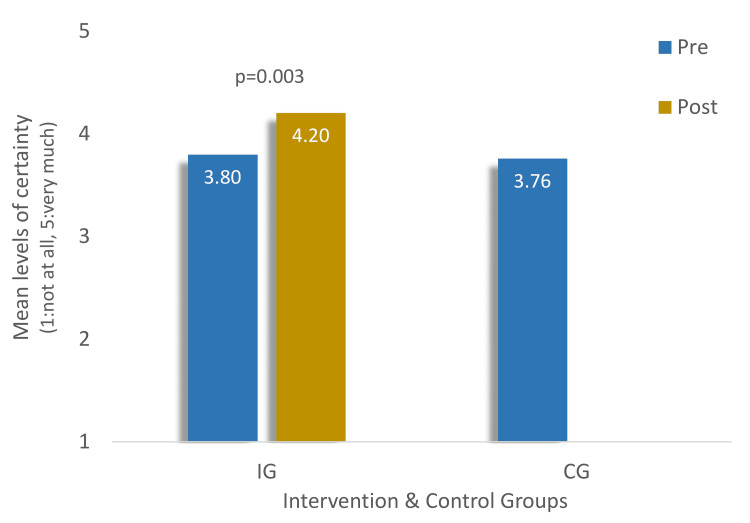
Levels of certainty of correct responses in knowledge, attitudes, and practices of the 34 physicians in the intervention and control groups IG - intervention group; CG - control group Mann-Whitney tests between IG and CG (p=0.919); Wilcoxon test in pre/post levels of IG (p=0.003)

As illustrated in Figure 1, the levels of response certainty regarding correct responses to questions were comparable between the two groups before the seminar, with scores of 3.8 and 3.76 for the IG and CG, respectively (p=0.919). Within the intervention group, there was a noteworthy improvement in the level of response certainty regarding correct responses among the 17 physicians, increasing from 3.8 to 4.2. This improvement yielded a statistically significant result (p=0.003).

As presented in Table 2, within the IG, thirteen out of sixteen questions seem to have improved scoring, while five questions seem to also show statistically significant variation. The three remaining questions, which exhibit a marginal decline in scores afterward, relate to two questions regarding the appropriate referral of a patient presenting with vertigo and one question addressing the proper need for referral of a patient with epistaxis. Questions Q1, and Q2 of the knowledge section achieved the greatest variability with pre- and post-scores of 41.2% to 100% and 23.5% to 100%, respectively.

**Table 2:** Frequency of correct responses for each knowledge, attitudes, and practices question among the 34 physicians in the intervention and control groups IG - intervention group; CG - control group McNemar test in the intervention group. ns: non-significant change (p>0.05). Q9 image can be seen in Figure 2.

Section (knowledge, attitudes, practices)	Questions and correct answers	Groups	Intervention		p-value
			Before	After	
			n (%)	n (%)	
Knowledge	Q1: In cases of anterior epistaxis the patient must:	IG	7 (41.2)	17 (100.0)	0.002
	A: Tilt the head forward and apply pressure at the anterior third of the nose for 5 minutes.	CG	8 (47.1)		
	Q2: In a patient with an active anterior epistaxis, the correct placement of the nasal packing device includes:	IG	4 (23.5)	17 (100.0)	<0.001
	A: Placement of lidocaine gel on the nasal pack device, positioning it horizontally with a backward direction, and then administration of normal saline	CG	7 (41.2)		
	Q3: During clinical examination Benign Paroxysmal Positional Vertigo usually:	IG	1 (5.9)	2 (11.8)	ns
	A: Does not present with spontaneous nystagmus and the Dix-Hallpike test is positive in the affected side	CG	4 (23.5)		
	Q4: In which cases a patient with vertigo must absolutely be referred to the Emergency Department? (correct answers might be more than one):	IG	4 (23.5)	3 (17.6)	ns
	A_1_: When he presents with vertical nystagmus A_2_: When he presents with third-degree spontaneous nystagmus	CG	6 (35.3)		
	Q5: The CENTOR criteria include the following except:	IG	7 (41.2)	13 (76.5)	0.031
	A: Non-tender cervical lymphadenopathy	CG	9 (52.9)		
	Q6: A patient suffering from a peritonsillar abscess usually presents with the following except one:	IG	13 (76.5)	17 (100.0)	ns
	A: Nasal congestion	CG	15 (88.2)		
	Q7: A patient with otitis externa should be treated with all the above except one:	IG	7 (41.2)	12 (70.6)	ns
	A: Administration of per os antibiotic treatment for 10 days	CG	9 (52.9)		
	Q8: What actions should be taken in a patient who suffers a hymenopter bite and presents with difficulty breathing and facial angioedema?	IG	10 (58.8)	13 (76.5)	ns
	A: Administration of intramuscular adrenaline 0.5mg	CG	10 (58.8)		
	Q9: What does the following image show (Figure 2):	IG	8 (47.1)	10 (58.8)	ns
	A: Acute otitis media	CG	14 (82.4)		
Attitudes	Q1: When do you believe that a patient with an active anterior epistaxis should be referred to the emergency department?	IG	16 (94.1)	13 (76.5)	ns
	A: In case of a failed attempt to control the bleed with a nasal packing device	CG	12 (70.6)		
	Q2: When do you believe that a patient with vertigo should be referred for immediate neurologic consult?	IG	17 (100.0)	16 (94.1)	ns
	A: In case of existing neurologic semiology	CG	17 (100.0)		
	Q3: When do you believe that a patient with otitis externa should be referred to the ED?	IG	7 (41.2)	8 (47.1)	ns
	A: When there is an abscess formation in the external ear canal	CG	9 (52.9)		
	Q4: In which cases do you believe that a patient with acute tonsilitis should be referred for immediate ENT consult? (correct answers might be more than one):	IG	1 (5.9)	7 (41.2)	0.031
	A_1_: In case of uvula displacement to one side A_2_: In case of coexisting trismus	CG	2 (11.8)		
Practices	Q1: In which of the following cases of a patient with upper respiratory infection do you administer antibiotic treatment in your daily practice?	IG	12 (70.6)	13 (76.5)	ns
	A: In a patient with fever >38 degrees Celsius, tonsillar exudate and swollen tender cervical lymphadenopathy without any other signs or symptoms	CG	10 (58.8)		
	Q2: In which of the following cases do you refer a patient with acute otitis media for emergency ENT consult in your everyday practice?	IG	8 (47.1)	14 (82.4)	0.031
	A: In case of tenderness and edema of the mastoid process	CG	6 (35.3)		
	Q3: In which of the following cases do you immediately refer a patient with epistaxis for ENT consult?	IG	8 (47.1)	10 (58.8)	ns
	A: In case of posterior bleeding after the placement of a nasal packing device	CG	5 (29.4)		

**Figure 2:**
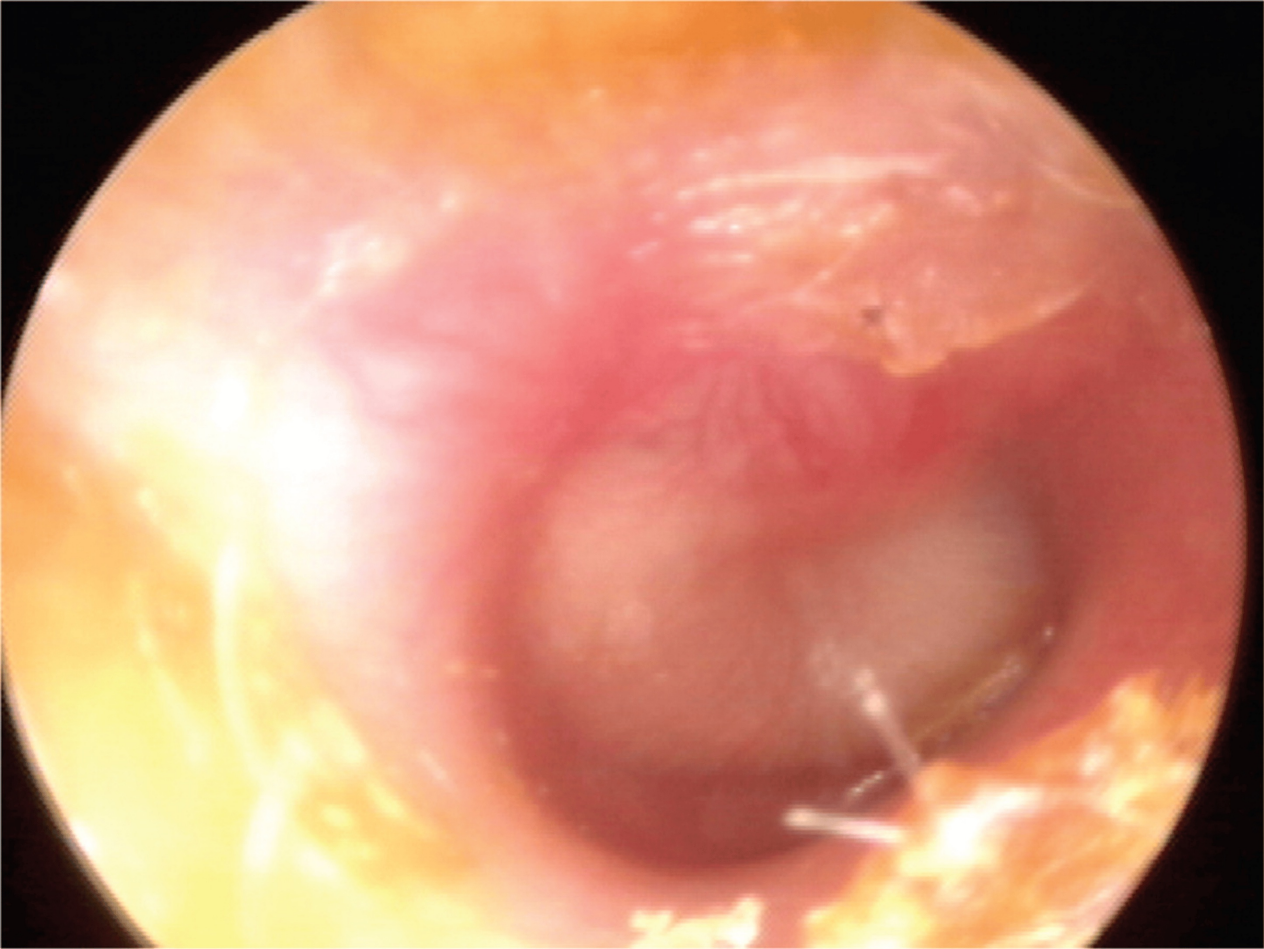
Image displayed in question Q9 of the knowledge section Otoscopic view of acute otitis media originated from reference [22] Figure 2 was reprinted with permission from Harmes KM, Blackwood RA, Burrows HL, Cooke JM, Harrison RV, Passamani PP: Otitis media: diagnosis and treatment. Am Fam Phys. 2013, 88:435-40 (c) 2013 American Academy of Family Physicians. All Rights Reserved.

As delineated in Table 3, the knowledge section documented the lowest scores, registering 39.9±20.8 for the IG and 53.6±19.7 for the CG, presenting a statistically significant difference between the groups. This variation was not observed to be significant when studying the "knowledge and attitudes", and all KAP domains together as the Δ-difference recorded was the smallest of +4.8 when all KAP domains were investigated, contrary to a +9.1 of the "knowledge and attitudes" and a +13.7 of the "knowledge" domains.

**Table 3:** Levels and changes in scores a of knowledge, attitudes, and practices among 34 physicians in intervention and control groups ^a^The score was determined in each section on the correct responses on a scale of 0-100 Knowledge - nine questions; Knowledge and attitudes - 13 questions; Knowledge, attitudes, and practices - 16 questions Mann-Whitney test was used.

Section scores		Total	Groups				p-value
			Intervention (n=17)		Control (n=17)		
			Mean±SD^ a^				
Knowledge	Baseline	47.7±21.1	39.9±20.8		53.6±19.7		0.038
	Δ-difference		13.7				
Knowledge and Attitudes	Baseline	50.7±17.1	46.2 ±16.3		55.1±17.3		0.14
	Δ- difference		9.1				
Knowledge, Attitudes, and Practices	Baseline	50.2±16.6	47.8±14.9		52.6±18.2		0.375
	Δ- difference		4.8				

When focusing on the IG's improvement in scoring presented in Table 4, statistical significance was recorded through all domains (p<0.05). Specifically, for the "knowledge" domain, the Δ-difference was +28.1 with an effect size of 0.81, and for the "knowledge and attitudes" +20.8 and 0.77, respectively. Finally, when all KAP domains were reviewed, a Δ-difference of +20.2 and an effect size of 0.85 was recorded.

**Table 4:** Levels of knowledge, attitudes, and practices scores before and after the intervention (educational course) among the 17 participating physicians in the study ^a^The score was determined in each section on the correct responses on a scale of 0-100 Knowledge - nine questions; Knowledge and attitudes - 13 questions; Knowledge, Attitudes, and Practices - 16 questions Wilcoxon tests were used. Effect size or |r| was estimated based on the formula of r=Z/√n.

Section scores			Intervention group			
			mean±SD^a^	Δ-difference	p-value	Effect size
Knowledge		Before	39.9±20.8	28.1	0.001	0.81
		After	68.0±14.6			
Knowledge and Attitudes		Before	46.2±16.3	20.8	0.001	0.77
		After	67.0±14.3			
Knowledge, Attitudes, and Practices		Before	47.8±15.0	20.2	<0.001	0.85
		After	68.0±13.2			

## Discussion

Questionnaire's completion

Both groups had a similar response in all domain performance, achieving scores of 47.8 and 52.6 out of 100 for the IG and CG, respectively. Although the observed disparity in knowledge scores between the two groups is statistically significant, differences in correct answers across all three sections lack statistical difference. In regards to knowledge scores and the similar feature composition among groups, this difference may be partially attributed to the reception conditions where the participants completed the questionnaire upon their arrival, in 'conditions of exams', at a different setting, since GPs from the CG completed the questionnaire in their medical office, which is a familiar environment to them. In addition, the knowledge section of the questionnaire may have created an inevitable impression of a testing procedure rather than a survey, prompting them possibly to some level of adaptation effort spending rather than an automatic knowledge recalling answer. This could also explain the time spent by IG participants to complete the questionnaire just before the seminar, in comparison to CG. Sections of attitudes and practices refer mostly to beliefs and not to knowledge testing. Nonetheless, the overall average starting performance, with a median of 50.2, suggests the need for continuous educational alertness, especially for clinical topics of high prevalence or vigilance. In 2020, a national study reporting on the educational needs of GP residents described the overall educational gain during residency, which relates to the needs and effort necessary to meet residents' expectations [26]. Moreover, a similar study reporting on a small educational course for training GPs on bronchial asthma in 2001 showed that the then-recent graduates could be educated with more favorable results than the qualified practitioners [27]. These findings may explain why periodically revisiting training goals and needs may be vital to the medical education continuum that doctors need and deserve.

As delineated in Table 1, participants were questioned on the tenure of English speaking language and international work experience. These two determinants may theoretically upgrade the ability to approach clinical problems by searching for evidence and recalling gained experience from abroad. A study by Edwardson et al. explained that some benefits of working abroad include helping to expand medical knowledge, improve physical examination skills, and advance procedural skills [28].

Furthermore, Table 2 elucidates the accurate responses extracted from the questionnaires of both study groups. Within the IG, a statistically significant improvement in responses to five out of sixteen questions was observed following the educational seminar (p<0.05). Two questions related to the management of epistaxis, two related to the diagnosis and management of acute tonsillitis (upper respiratory infection), and one addressed the management of acute otitis media (AOM). These findings suggest that GPs exhibited greater responsiveness to the seminar in managing and diagnosing the above-mentioned cases.

Specifically, epistaxis is frequently encountered by GPs as it has been reported to be extremely common [24]. The questions regarding epistaxis (Q1 and Q2 "knowledge") pertained to the initial management and the correct placement of the nasal packing. Their practical nature and ease of assimilation probably led all participants to answer correctly after the seminar. The questions and presentations were made according to existing guidelines addressed not specifically to qualified ENT specialists but also to primary care providers [11]. The questions about URIs Q5 "knowledge" and Q4 "attitudes" were focused on diagnosing the signs of a peritonsillar abscess in need of an immediate referral to the ED and the management of acute tonsilitis. The CENTOR criteria constitute a validated clinical decision rule set that is easily applicable by the clinician in order to determine the proper management of acute tonsilitis [18], while the peritonsillar abscess is most commonly a complication of streptococcal tonsilitis [19]. Finally, question Q2 "practices", regarded the need for proper referral of AOM cases to the ED for immediate ENT consult. AOM is a common problem amongst pediatric population groups [22], and mastoiditis presenting as a complication or progression of AOM is a life-threatening condition that physicians need to possess the ability to correctly identify [25]. Since GPs of the region are often required to examine children, diagnosing and properly treating AOM, as well as shaping opinion on referral necessity, is fundamental, and appropriate training in these cases could positively influence patient care.

On the contrary, conditions such as vertigo and benign paroxysmal positional vertigo (BPPV) appeared to pose more dilemmas for GPs in terms of diagnosis and treatment, both pre and post-intervention. In particular, questions Q3 "knowledge" regarding the diagnosis of BPPV, while Q2 "attitudes" and Q4 "knowledge" regarding the immediate referral of vertigo to the ED for a consult. These questions yielded interesting results, as the scores in the knowledge section remained low even after the seminar, while the participants scored very well in the attitudes section. Both questions regarding referrals highlight the urgency of patients presenting with neurologic deficits, indicating an underlying neurologic condition or signs of vestibular neuritis, as these patients might need additional imaging or even admission [14-18]. However, even though participants excelled in the "attitudes" questions, they underscored in the "knowledge" section. This can primarily be attributed to the difficulty of question Q4 "knowledge" about referring a patient with vertigo to the ED, in which there was more than one correct answer, thus troubling the participants.

Overall, GPs seem to harbor a lack of confidence in treating or safely diagnosing BPPV, potentially stemming from concerns about misdiagnosis and the associated risk of severe complications related to a neurologic condition such as a stroke. Some conditioned 'neurophobia' among residents has been detected in a national study conducted recently [27]. GP residents were surveyed about their training satisfaction and expressed their need to further enhance their experience by rotating neurology departments [26]. Furthermore, a study conducted in 2019 suggested some pragmatic weaknesses met by the GPs in performing diagnostic and canalith repositioning maneuvers in patients suspected of having BPPV, with low frequencies of skill practicing [29].

The scores in knowledge, attitudes, and practices sections within the IG demonstrate a significant improvement before and after the seminar, as outlined in Table 4. Notably, there was a statistically significant enhancement with substantial effect sizes observed in all KAP sections. Moreover, the effect size appears larger in the "KAP" domain, suggesting a higher efficiency in variation rather than in each domain individually. This significant improvement across all sections depicts an 'experimental viability' of the seminar in enhancing GPs' overall competency, added to the improvement in regards to the levels of response certainty shown in Figure 1. Our findings align with existing literature and further support the perception that tolerably short-duration seminars can effectively enhance the overall performance of participants [27,30].

Course organization

In the field of continuous medical education, numerous courses with diverse training approaches exist. Nevertheless, organizing such programs, particularly for seasoned physicians, presents distinctive challenges. Based on the authors' personal experience, during the process of approaching the GPs to attend the course, we realized that there was a higher chance of getting participation consent when the invitation was made in person and not via e-mail or phone call. Many GPs agreed to attend the seminar upon being informed that other colleagues who they were familiar with had confirmed their availability as well. Additionally, the short three-hour duration of the seminar with the context of five lectures with few minute breaks in between was supported by existing literature and was appealing and appreciated by the physicians who ended up attending the seminar [31].

On the other hand, many were reluctant to attend due to the seminar being held on a Saturday, as it is their day off. Also, concerns were raised by some participants about the anonymity of the questionnaires, which were addressed by creating a 'username' for pre- and post-completion comparisons, by using a self-selected date and city, freely chosen by each participant. This allowed the identification of the individual questionnaires before and after intervention in order to assess trends of variation while guaranteeing the participants' anonymity in the process. However, despite our best efforts, the primary limitation faced in recruiting participants was the inertia of the GPs to attend the course, since from 103 people in total, 51 were willing to participate, with 34 finally randomly included. This reluctance has been met within other local studies in the past, as in a Cretan educational seminar many years ago from the 14 health centers of the island, only 21 medical professionals agreed to attend [27].

Strengths and limitations

First of all, our intervention group represented just 16.5% of the GPs practicing in the county of Heraklion. Secondly, the two group participants completed the questionnaires in different settings. The intervention group participants may have initially perceived some sense of feeling less 'comfort', a reason that was translated to a delay and slight embarrassment about their performance when initiated to complete the pre-intervention questionnaire. There was some difference between the ability to speak English in the two groups. We cannot estimate the cumulative net impact of this difference on the baseline responses since the control group with less English knowledge showed a reversed trend performance at the initial moment. Additionally, the seminar was exclusively performed in the Greek language, as all participants were native Greeks.

The educational themes have been selected and tailored from local epidemiologic studies to address the morbidity by reflecting an evidence-based structuring of an educational course with community clinical orientation. Furthermore, apart from the presentations, audiovisual files were used as well to make the educational material more easily assimilated. The seminar was of low cost, enough to prove that similar educational initiatives can be afforded without funding available. Last but not least, overall satisfaction was verbally reported high, with no complaints, at the end of the seminar, although a thought that occurred afterward was that a satisfaction scale might be included within similar initiatives, especially when larger groups do not allow post-seminar direct interaction.

## Conclusions

This compact three-hour intensive ENT educational course, based on prevalent ENT morbidity originating from existing literature and local epidemiologic data, exhibited significant usefulness in producing pre- and post-variation in terms of confidence and response improvement. Although attendance to the seminar was limited, one-third of GPs working at the county participated. The findings strongly advocate for the systematic integration of such targeted short-duration courses into ongoing GP education, emphasizing the delivery of practical and, if possible, locally relevant content to bridge eventual practicing gaps. The organization of educational seminars for senior physicians, despite their challenging nature, can be accomplished by implementing tested frameworks and multidisciplinary design. Future studies should explore the long-term effects of short educational courses and better address 'formats' for continuous education programs within primary care, with ingredients such as usefulness, cost-effectiveness, and feasibility.
